# HIV, HBV and HCV Coinfection Prevalence in Iran - A Systematic Review and Meta-Analysis

**DOI:** 10.1371/journal.pone.0151946

**Published:** 2016-03-31

**Authors:** Fahimeh Bagheri Amiri, Ehsan Mostafavi, Ali Mirzazadeh

**Affiliations:** 1 Department of Epidemiology, Pasteur Institute of Iran, Tehran, Iran; 2 Department of Epidemiology, University of Tehran, Tehran, Iran; 3 Research Centre for Emerging and Reemerging infectious diseases, Pasteur Institute of Iran, Akanlu, Kabudar Ahang, Hamadan, Iran; 4 Department of Epidemiology and Biostatistics, University of California, San Francisco, CA, United States of America; 5 Regional Knowledge Hub, and WHO Collaborating Centre for HIV Surveillance, Institute for Futures Studies in Health, Kerman University of Medical Sciences, Kerman, Iran; Defence Research Laboratory, INDIA

## Abstract

**Background:**

worldwide, hepatitis C and B virus infections (HCV and HCV), are the two most common coinfections with human immunodeficiency virus (HIV) and has become a major threat to the survival of HIV-infected persons. The review aimed to estimate the prevalence of HIV, HBV, HCV, HIV/HCV and HIV/HBV and triple coinfections in different subpopulations in Iran.

**Method:**

Following PRISMA guidelines, we conducted a systematic review and meta-analysis of reports on prevalence of HIV, HBV, HCV and HIV coinfections in different subpopulations in Iran. We systematically reviewed the literature to identify eligible studies from January 1996 to March 2012 in English or Persian/Farsi databases. We extracted the prevalence of HIV antibodies (diagnosed by Elisa confirmed with Western Blot test), HCV antibodies and HBsAg (with confirmatory laboratory test) as the main primary outcome. We reported the prevalence of the three infections and coinfections as point and 95% confidence intervals.

**Findings:**

HIV prevalence varied from %0.00 (95% CI: 0.00–0.003) in the general population to %17.25 (95% CI: 2.94–31.57) in people who inject drugs (PWID). HBV prevalence ranged from % 0.00 (95% CI: 0.00–7.87) in health care workers to % 30.9 (95% CI: 27.88–33.92) in PWID. HCV prevalence ranged from %0.19 (95% CI: 0.00–0.66) in health care workers to %51.46 (95% CI: 34.30–68.62) in PWID. The coinfection of HIV/HBV and also HIV/HCV in the general population and in health care workers was zero, while the most common coinfections were HIV/HCV (10.95%), HIV/HBV (1.88%) and triple infections (1.25%) in PWID.

**Conclusions:**

We found that PWID are severely and disproportionately affected by HIV and the other two infections, HCV and HBV. Screenings of such coinfections need to be reinforced to prevent new infections and also reduce further transmission in their community and to others.

## Introduction

HIV and viral hepatitis infections are still the major causes of morbidity and mortality in developing countries, with one billion people directly exposed or at-risk population [1,2]. Worldwide, 34 million people are infected with HIV, 130 million people are infected with HCV (Hepatitis C Virus), 2 billion people are infected with HBV (Hepatitis B Virus), and 350–400 million people are suffering from viral chronic hepatitis (4–7). Annually, approximately two million people die due to AIDS, more than 350 thousands people die from diseases associated with HCV and one million people die as a result of an HBV infection [3–5].

In Iran, the prevalence of HIV and other blood-borne viral infections like HCV is relatively low in the general population[6]. Prevention strategies like public awareness on routes of transmission, free HIV testing and counseling services at public health facilities and correctional institutes like prisoners contributed to this low prevalence. Screening for HCV and HIV in all blood donors and all blood products have been in place since 1996 and 1989 respectively. Countrywide harm reduction services including, but not limited to needle exchange programs (delivered by 682 centers) and drug treatments like methadone maintenance therapy (delivered by 4275 centers) have been implemented by governmental funds [7]. The government of Iran is committed to provide universal access to HIV prevention (free condom, education, HIV testing) and antiviral therapy services for all at-risk or affected populations as outlined in the 4^th^ National AIDS Strategic Plan 2015–19[8]. These heath policies and interventions aim to reduce the burden of main blood-borne infections in Iran.

HCV and HIV share common transmission risk behaviors, either monoinfection or HCV/HIV coinfection have been reported in population of drug injectors worldwide (10). Despite HIV and HBV, sexually-acquired or vertical transmitted HCV is not common [9]. These coinfections could lead to accelerated chronic hepatitis and liver cancer (11), which reported as one of the major causes of morbidity and mortality in HIV-infected individuals (12). The most affected population are PWID (13).

The HIV epidemic in Iran is concentrated among PWID with the pooled HIV prevalence of 18.4% (95% CI: 16.7, 20.2) after 2005[10]. HIV has been in the radar of national AIDS prevention and treatment programs. The trends of HIV and risk behaviors have been studied in several national bio-behavioral surveys [11,12]. However, screening for HCV in HIV-infected patients [13] and annual screening in high-risk population like PWID, as recommendations by guidelines [14], has not been implemented systematically. One reason is that the scope of HCV and HBV coinfections with HIV has not been studied in Iran.

Many subnational studies have assessed HIV and HCV coinfections, mostly among people who inject drugs [15–20], and prisoners [21–25], however the overall size of such co-epidemics is unknown in Iran.

### Objectives

In this systematic review, we aimed to estimate the prevalence of HIV, HCV and HBV infection and HIV coinfections and identify the most affected subpopulations in Iran.

## Methods

### Information sources and search

From January 1996 to March 2012, we searched the literature for articles that assessed the prevalence of HIV, HBV or HCV infection and coinfections.

Between March and June 2012 we searched multiple English and Persian/Farsi electronic data sources including Pubmed, Iranmedex, Google Scholar, Iranian Data Bank of Hepatitis Research, Iranian Data Bank of HIV Research, Scientific Information Database (SID), Magiran and the Iran Blood Transfusion Journal. We reviewed the titles and abstracts to select potentially relevant papers. If there was doubt about the suitability of the paper based on the abstract, the full text was reviewed. We manually searched the references and relevant articles for inclusion. We also looked at the electronic abstract list of congresses conducted in Iran and also at the electronic database of students’ thesis through universities” electronic libraries and websites, when was available.

Keywords that we used for our search were “HIV and HBV”, “HIV and HCV”, “viral hepatitis and HIV” and “coinfection and HIV”. Studies that reported HIV infection as well as HBV or HCV were included.

### Eligibility criteria and study selection

Only studies that recruited participants living in Iran, published in Persian/Farsi or English, measured HIV (HIV Ab) and coinfections such as HBV (HBs Ag) or HCV (HCV Ab) infection with confirmatory lab tests were included. We excluded studies with 1) no accessible full text and no sufficient data in abstract, 2) unclear serological tests to detect the three infections, 3) low quality due to incorrect reporting of prevalence and/or an unclear number of cases with a positive test and 4) reported viral hepatitis prevalence only in HIV-positive individuals (i.e. only HIV positive cases were recruited in the study).

We contacted the corresponding author when we have questions about the eligibility of the article or a critical data was missing.

### Data collection and data items

Data was extracted by one reviewer and double checked for the following items: type of study, sample size, location and time of the study, type of participants and prevalence of HIV, HBV and HCV and the coinfections. We grouped the participants into six subpopulations 1) patients with hemophilia, thalassemia or hemodialysis (patients who received multiple transfusions), 2) people who inject drugs, 3) prisoners, 4) general population (pregnant women, blood donors and cadavers with low risk), 5) health care workers, and 6) street children.

### Analytic Approach

We conducted meta-analyses in STATA version 11. We did meta-analysis for each HIV, HCV, HBV, HIV/HBV, HIV/HCV and HIV/HBV/HCV prevalence in every subpopulation, pending on the data availability. The outcome was reported as prevalence, with point and 95% confidence intervals. A Q-test was used to assess heterogeneity. When the heterogeneity test was statistically significant (p-value < 0.1), a random-effects model was used; otherwise the fixed-effects model was applied to calculate the pooled prevalence.

## Results

### Search Results

**Fig 1:**
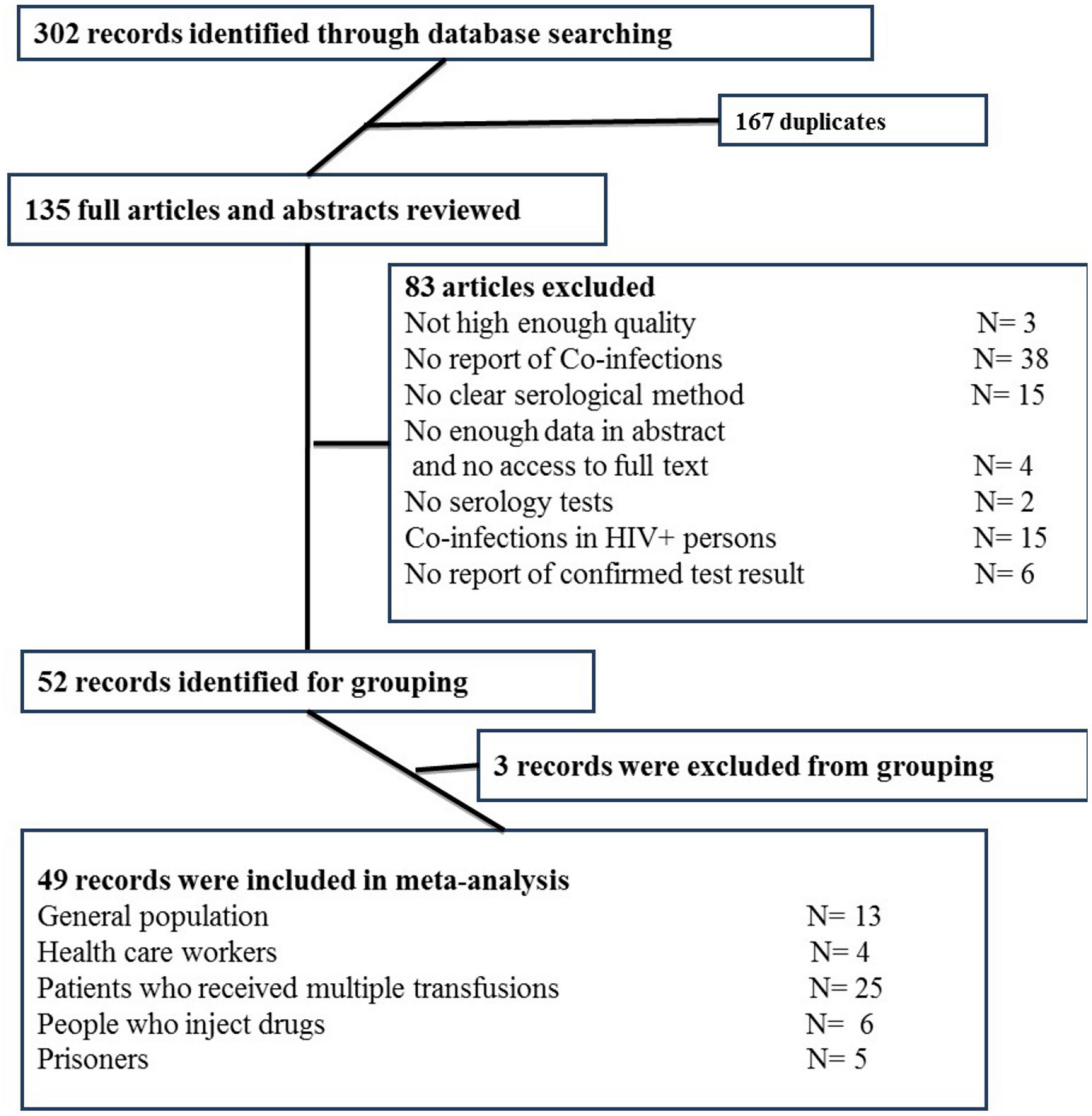
Flow diagram of included and excluded records.

As presented in Fig 1, we found 302 abstracts in our literature review. After removing duplications (167) based on title and abstract, 135 remained for fulltext review. Of those, 83 articles were excluded (S1 Table) for various reasons including poor quality (3), no report of coinfections (38), unclear serologic tests (15), fulltext was not available (4), no serology test (2), only recruited HIV positive cases (15) no confirmatory lab test results (6), and specific subpopulations not listed in the six population categories we have chosen as priori (3).

Overall, more than 170,000 individuals (170,378 from general population) have been recruited in the 49 included studies. Most of the studies (25 out of 49) were conducted among patients who received multiple transfusions (PWRMT), followed by general population (13). The summary of included studies is presented in the Table 1.

**Table 1:** Characteristics of the included studies in the systematic review.

	Group	Sample Size	Study site	Reference
1	Blood donors	7997	Isfahan	[37]
2	Blood donors	20294	Bushehr	[38]
3	Blood donors	38920	Gorgan	[39]
4	Blood donors	23716	Isfahan	[40]
5	Blood donors	1208	Qom	[41]
6	Blood donors	42652	South Khorasan	[42]
7	Blood donors	4808	Isfahan	[43]
8	Blood donors	12935	Qom	[44]
9	Blood donors	4980	Yasuj	[45]
10	Blood donors	400	Khuzestan	[46]
11	Blood donors	11615	Arak	[47]
12	Pregnant women	680	Malekan	[48]
13	Cadaver of low risk persons	173	Tehran	[49]
14	Health care workers	110	Kermanshah	[50]
15	Health care workers	328	Kermanshah	[51]
16	Health care workers	112	Tehran	[52]
17	Dentists	45	Kashan	[53]
18	Hemodialysis patients	324	Tabriz	[54]
19	Hemophilic patients	176	Tehran	[55]
20	Hemophilic patients	553	Isfahan	[56]
21	Hemophilic patients	80	Birjand	[57]
22	Hemophilic patients	74	Yazd	[58]
	Thalassemia patient	85		
23	Persons with Combined Factor V & VIII deficiency	24	Mashhad	[59]
24	Thalassemia patient	360	Mashhad	[60]
25	Thalassemia patient	84	Tabriz	[61]
26	Thalassemia patient	97	Markazi	[62]
	Hemophilic patient	68		
27	Thalassemia patient	121	Tehran	[63]
28	Thalassemia patient	616	Isfahan	[64]
	Hemophilic patient	120		
29	Thalassemia patient	142	Qom	[65]
30	Thalassemia patient	732	Tehran, Kerman, Qazvin, Semnan, Zanjan	[66]
31	Thalassemia patient	95	Qazvin	[67]
32	Thalassemia patient	122	Ahwaz	[68]
33	Thalassemia patient	206	Ahwaz	[69]
34	Thalassemia patient	545	Isfahan	[70]
	Hemophilic patient	615	Isfahan	[70]
35	Thalassemia patient	50	Hamedan	[71]
36	Intravenous drug users	417	Tehran	[17]
37	Intravenous drug users	153	Arak	[16]
38	Intravenous drug users	92	Isfahan	[18]
39	Intravenous drug users	240	Sanandaj	[20]
40	Intravenous drug users	899	Tehran	[19]
41	Intravenous drug users	88	Tehran	[15]
42	Prisoners	346	Zanjan	[23]
43	Prisoners	252	Bandar Abbas	[25]
44	Prisoners	1431	Isfahan, Chaharmahal Bakhtiary, Lorestan	[21]
45	Prisoners	358	Birjand	[22]
46	Prisoners	400	Birjand	[24]
47	Street children	102	Tehran	[35]
48	Street children	386	Isfahan	[36]
49	Hemodialysis patients	104	Bushehr	[72]
	Thalssemia patient	355	Bushehr	[72]
	Intravenous drug users	19	Bushehr	[72]
	Hemophilic patients	31	Bushehr	[72]

The prevalence of HIV, HBV and HCV and different combination of such infections are presented in Table 2.

**Table 2:** Prevalence of different infections and their co-morbidity among different Iranian groups, 1996–2012.

Outcome	Group	Sample size	Number of study	Pooled estimate (%)
**HIV**	GP	170378	13	0.00(0.00–0.003)
	HCW	595	4	0.00(0.00–0.50)
	PWRMT	5779	25	0.01(0.00–0.12)
	Street children	488	2	0.00 (0.00–0.46)
	Prisoner	2787	5	3.42(1.22–5.63)
	PWID	921	5	17.25(2.94–31.57)
**HBV**	GP	170378	13	0.90 (0.57–1.22)
	HCW	45	1	0.00(0.00–7.87)
	PWRMT	142	1	0.70 (0.00–2.07)
	PWID	899	1	30.90 (27.88–33.92)
**HCV**	GP	170378	13	0.31 (0.18–0.43)
	HCW	595	4	0.19 (0.00–0.66)
	PWRMT	3919	19	19.28(13.98–24.56)
	Street children	488	2	0.76 (0.00–1.62)
	Prisoner	1431	1	34.73 (32.26–37.20)
	PWID	1403	5	51.46(34.30–68.62)
**HIV/HBV**	GP	170378	13	0.00(0.00–0.003)
	HCW	595	4	0.00(0.00–0.50)
	PWRMT	5779	25	0.00(0.00–0.11)
	Prisoner	2787	5	0.13(0.00–0.42)
	PWID	172	2	1.88(0.00–4.03)
**HIV/HCV**	GP	170378	13	0.00(0.00–0.003)
	HCW	595	4	0.00(0.00–0.50)
	PWRMT	5779	25	0.01(0.00–0.12)
	Street children	488	2	0.00 (0.00–0.46)
	Prisoner	2787	5	1.71(0.11–3.30)
	PWID	921	5	10.95(2.82–19.08)
**HIV/HBV/HCV**	GP	170378	13	0.00(0.00–0.003)
	HCW	595	4	0.00(0.00–0.50)
	PWRMT	5779	25	0.01(0.00–0.12)
	Prisoner	2787	5	0.28(0.00–0.58)
	PWID	172	2	1.25(0.00–3.01)

GP: General population; HCW: Health care worker; PWID: People who inject drugs; PWRMT: Patients who received multiple transfusions

#### HIV infection

The HIV prevalence in different subgroups varied from %0.00 (95% CI: 0.00–0.003) in the general population to %17.25 (95% CI: 2.94–31.57) in PWID. Prisoners were the second affected group, following PWID, with HIV prevalence as high as 3.42% (95% CI: 1.22–5.63). HIV prevalence in other groups was very low and close to zero.

#### HBV infection

The prevalence of HBV varied from %0.00 (95% CI: 0.00–7.87) in health care workers to the highest level of %30.9 (95% CI: 27.88–33.92) in PWID. HBV prevalence in the general population was close to 1%, and in PWRMT near to 0.7%.

#### HCV infection

The prevalence of HCV was surprisingly high among PWID as 51.46% (95% CI: 34.30–68.62). In addition, 34.73% of prisoners diagnosed with HCV, followed by 19.28% in PWRMT. HCV was assessed among street children in two studies with an estimated prevalence of 0.76%, considerably higher than the prevalence in the general population (0.31%).

#### HIV and HBV coinfection

Prevalence of both HBV and HIV in the general population, health care workers and PWRMT was very low and close to 0%. The highest prevalence of HIV/HBV coinfection was observed among injecting drug users as 1.88% (95% CI: 0.00–4.03). Among the prisoners, the HIV/HBV prevalence (0.13%) was lower than the PWID group but more than the other three subpopulations.

#### HIV and HCV coinfection

The HIV/HCV coinfection prevalence was very low and close to zero among the general population, health care workers and street children. However, 10.95% (95%CI: 2.82–19.08%) of PWID were positive for both HIV and HCV. In compare to general population, prisoners had also a higher prevalence of HIV/HCV (1.71%, 95%CI 0.11–3.30).

#### HCV/HBV/HIV coinfection

The prevalence of such triple coinfections was very low and close to zero in the general population, health care workers and street children; while it peaked to %1.25 (95% CI: 0.00–3.01) in PWID. PWRMT and prisoners had a low prevalence as 0.01% and 0.28%, not statistically different from the prevalence in the general population.

## Discussion and Conclusion

The results of our study showed that in people who inject drugs, one in six infected with HIV, one in two infected with HCV and one in three was seropositive for HBV. Our findings indicated that one in ten of people who inject drug in Iran are infected with both HIV and HCV.

Ye et al, in their recent review paper, reported HIV-HCV coinfection as 34.1–98.5% in countries of South and Southeast Asia. They included studies that recruited HIV positive cases (both conditional and joint probabilities) [26], but we did not. Although our estimate is in range of what they reported, our estimate would have been much higher if we included also studies that recruited only HIV positive cases. In another word, we are reporting the joint probability of HIV-HCV, but they have mixed both conditional and joint probabilities. For example, in one article that we excluded, 391 HIV positive cases were assessed for their drug use behaviors and biomarkers of HCV, and 172 (43.9%) were HCV positive [27]. Researchers need to clearly and accurately define what measure they are reporting when talking about coinfections.

HIV and HCV both transmitted through body fluids and blood; both can infect patients for years before even symptoms are manifested; currently what we observed in Iran is that the health sector has been given so much attention to HIV, and neglected curable co-morbidities like HCV. It has a payoff, irreversible hepatocirrhosis and hepatocarcinoma in near future of undiagnosed or miss-managed cases [28] and further transmission of HCV in the community, mostly injecting drug users.

This review highlighted the existing and seriousness of HCV and HIV-HCV joint infection particularly in those who inject drugs and the related key population, prisoners in Iran. In Iran, considerable number of PWID have reported risk factor for HIV and HIV-HCV coinfections such having incarceration history, shared injection inside and outside of prison, history of being tattooed inside prison, and sex work for drug or money [29–32]. Likewise among prisoners, the history of unsafe injection (28%), history of heterosexual sex (28%) and homosexual contacts (8%) are common, as long as tattooing which was reported by 51% incarcerated participants[21]. In both recent (2009 and 2014) national surveys among prisoners, dried blood samples were examined for HIV, not HCV; a missed opportunity to study the risk factors and the scope of HCV among the second affected population in Iran, prisoners[12].

We found that patients who received multiple transfusions have the same prevalence of HIV, HB and HIV-HBV coinfections as general population. This is an assuring finding that the screening of the blood products in Iran is effective for such infections. The Blood Transfusion System of Iran is applying a two-stage screening: asking about the high-risk behaviors of donors and performing routine tests on donated blood for HBs Ag (since 1974), HIV (since 1989) and HCV (since 1996) (28). However, although blood products have been screened for HCV, surprisingly we found that one out of five of patients with multiple blood transfusions, infected with HCV. Some of the articles (Ref 60, 50) that we included were conducted before 1996, when there was no nationwide HCV screening for blood product in place. In other two papers, participants received blood transfusion more than 10 years (Ref 61) and with high frequency of transfusion (Ref 62). High prevalence of HCV in thalassemia patients has been reported in Pakistan (40%) (29) and Egypt (76%) (30), but both have a very high level of HCV prevalence in the general population, which is not the case in Iran.

Prevalence of all three types of infections in health care workers was lower than other at risk groups. This could be due to their high level of knowledge, positive attitude and practice regarding the transmission of these infections [33,34] and an indication of safe practices and so limited chance of occupational hazardous transmission.

General population category mainly included studies on blood donors. People who wants to donate blood in Iran will go through a screening process of past exposures including counseling for sex and injection risk behaviors (33). Only those with no history of such exposures are allowed to donate blood. Assuming that most of the donors report correctly their risk exposures, our observed prevalence for HIV and other coinfections may be underestimated for the general population.

We found two surveys conducted among street children. The prevalence of HIV was zero and HCV prevalence was about 1%. None of the participants in the two studies have reported sexual contacts or injections [35,36]. Two possible explanations are either they have been borne as HCV positive (got the infection from their mothers) or they did not report or recall correctly their risk behaviors.

We did not find any studies on two important populations, female sex workers and men who have sex with men; although the prevalence of monoinfections among them have been assessed in a few studies, none reported coinfections. This gap needs to be addressed by targeting these populations in the surveillance surveys of the national AIDS program in Iran.

Our findings have some limitations. We only include studies that have reported joint probabilities of coinfections. Age was reported very differently by the original studies, so we were not able to report the findings by age groups.

Conclusion: Our results highlighted the seriousness of viral hepatitis, particularly HCV, as a coinfection with HIV. Half of people who inject drug affected by HCV while one in four affected by HIV. Given the current HIV/HCV syndemicity in Iran, joint planning, surveillance, healthcare delivery, disease prevention, and clinical care delivery can help to reduce the burden of these infection in Iran more effectively.

## Supporting Information

S1 Table Excluded Articles and reasons of exclusion. (DOCX)

S2 Table PRISMA Checklist. (DOC)
