# Supplementary material for: HIV, HBV and HCV Coinfection Prevalence in Iran - A Systematic Review and Meta-Analysis
Source: PLoS One. 2016 Mar 31;11(3):e0151946. doi: 10.1371/journal.pone.0151946 (PMC4816272; doi:10.1371/journal.pone.0151946)
Supplement: S1 Table — (DOCX) [file pone.0151946.s001.docx]

**S1 Table. Excluded Articles and reasons of exclusion**

|  | Study | Reasons for exclusion |
| --- | --- | --- |
| 1 | Ramezani ,A [1] | They have recruited only HIV positive cases into the study. |
| 2 | Davarpanah, MA [2] | They have recruited only HIV positive cases into the study. |
| 3 | Alavi, SM [3] | They have recruited only HIV positive cases into the study. |
| 4 | Ramezani, A [4] | They have recruited only HIV positive cases into the study. |
| 5 | Babamahmoodi, F [5] | They have recruited only HIV positive cases into the study. |
| 6 | Taeri, K [6] | They have recruited only HIV positive cases into the study. |
| 7 | Mohammadi, M [7] | They have recruited only HIV positive cases into the study. |
| 8 | Ataei, B [8] | They have recruited only HIV positive cases into the study. |
| 9 | Sharif-Mood, B [9] | They have recruited only HIV positive cases into the study. |
| 10 | Moradmand Badie, B [10] | They have recruited only HIV positive cases into the study. |
| 11 | SeyedAlinaghi, SA [11] | They have recruited only HIV positive cases into the study. |
| 12 | Rezaianzadeh, A [12] | They have recruited only HIV positive cases into the study. |
| 13 | Rahimi, MK [13] | They have recruited only HIV positive cases into the study. |
| 14 | Bagheri, P [14] | They have recruited only HIV positive cases into the study. |
| 15 | Keshvari, M[15] | They have recruited only HIV positive cases into the study. |
| 16 | Moniri, R [16] | No Co-infection was reported. |
| 17 | Kasraian, L [17] | No Co-infection was reported. |
| 18 | AghaJaniPoor, K [18] | No Co-infection was reported. |
| 19 | Tahaei, SME [19] | No Co-infection was reported. |
| 20 | Alaoddolehei, H [20] | No Co-infection was reported. |
| 21 | MohammadAlizadeh, AH [21] | No Co-infection was reported. |
| 22 | Imani, SR [22] | No Co-infection was reported. |
| 23 | Mirahmadizadeh, AR[23] | No Co-infection was reported. |
| 24 | Attarchi, Z[24] | No Co-infection was reported. |
| 25 | Mahmoodian-Shooshtari, M [25] | No Co-infection was reported. |
| 26 | Daee‌Parizi, Z [26] | No Co-infection was reported. |
| 27 | Emmam, SJ [27] | No Co-infection was reported. |
| 28 | Khedmat, H [28] | No Co-infection was reported. |
| 29 | Khodadadizadeh, A [29] | No Co-infection was reported. |
| 30 | Khomeisipour, GR [30]** | No Co-infection was reported. |
| 31 | Kolivand, M [31] | No Co-infection was reported. |
| 32 | Tofighi, H [32] | No Co-infection was reported. |
| 33 | Torabi, SA [33] | No Co-infection was reported. |
| 34 | Rezazadeh, M [34] | No Co-infection was reported. |
| 35 | Rowhani-Rahbar, A [35] | No Co-infection was reported. |
| 36 | NourKojory, S [36] | No Co-infection was reported. |
| 37 | Alavi, SM [37] | No Co-infection was reported. |
| 38 | Taheri Azbarmi, Z [38] | No Co-infection was reported. |
| 39 | Samar, G [39] | No Co-infection was reported. |
| 40 | Soudbakhsh, AR [40] | No Co-infection was reported. |
| 41 | Ebrahimian, Z [41] | No Co-infection was reported. |
| 42 | Shariatzadeh, SMA [42] | No Co-infection was reported. |
| 43 | Bani aghil, SS [43] | No Co-infection was reported. |
| 44 | [Zamani, S](http://www.ncbi.nlm.nih.gov/pubmed?term=Zamani%20S%5BAuthor%5D&cauthor=true&cauthor_uid=20483578) [44] | No Co-infection was reported. |
| 45 | Mirahmadzadeh, AR [45] | No Co-infection was reported. |
| 46 | Abdollahi, A [46] | No Co-infection was reported. |
| 47 | Jahani, M [47] | No Co-infection was reported. |
| 48 | Pourazar, A [48]** | No Co-infection was reported. |
| 49 | Karimi, M [49] | No Co-infection was reported. |
| 50 | Aghakhani, A [50] | No Co-infection was reported. |
| 51 | Mahmoudian Shoushtari, M [51] | No Co-infection was reported. |
| 52 | Amini Kafi abad, S [52] | No Co-infection was reported. |
| 53 | Esmaeili, H [53] | No Co-infection was reported. |
| 54 | Khoshnood mansoorkhani, MJ [54] | The type of the serology test was not clearly defined in the paper. |
| 55 | Hajinasrollah, E [55] | The type of the serology test was not clearly defined in the paper. |
| 56 | Tabatabaee, S [56] | The type of the serology test was not clearly defined in the paper. |
| 57 | Azarkeivan, A [57] | The type of the serology test was not clearly defined in the paper. |
| 58 | Moradi, A [58] | The type of the serology test was not clearly defined in the paper. |
| 59 | Emamghorashi, F [59] | The type of the serology test was not clearly defined in the paper. |
| 60 | Sayadjoo, S[60] | The type of the serology test was not clearly defined in the paper. |
| 61 | Kazeroni, P [61] | The type of the serology test was not clearly defined in the paper. |
| 62 | Ghanbarzadeh, N [62] | The type of the serology test was not clearly defined in the paper. |
| 63 | Kasraian, L [63] | The type of the serology test was not clearly defined in the paper. |
| 64 | Kazerani, H [64] | The type of the serology test was not clearly defined in the paper. |
| 65 | Sarveghad, MR [65] | The type of the serology test was not clearly defined in the paper. |
| 66 | Shariat, SV [66] | The type of the serology test was not clearly defined in the paper. |
| 67 | Sharifi-Mood, B [67] | The type of the serology test was not clearly defined in the paper. |
| 68 | Bakhshi, Sh [68] | The type of the serology test was not clearly defined in the paper. |
| 69 | Meidani, M [69] | The results from the serology test were not confirmed by another confirmatory test. |
| 70 | Aminzadeh, Z [70] | The results from the serology test were not confirmed by another confirmatory test. |
| 71 | [Sharif, M](http://www.ncbi.nlm.nih.gov/sites/entrez?cmd=search&db=PubMed&term=%20Sharif%2BM%5bauth%5d) [71] | The results from the serology test were not confirmed by another confirmatory test. |
| 72 | Keramat, F [72] | The results from the serology test were not confirmed by another confirmatory test. |
| 73 | Vahdani, P [73] | The results from the serology test were not confirmed by another confirmatory test. |
| 74 | Mir-Nasseri, MM [74] | The results from the serology test were not confirmed by another confirmatory test. |
| 75 | Bagheri Mahaki, E [75] | The full text was not available. |
| 76 | Salari, Z [76] | The full text was not available. |
| 77 | Beheshti, M [77] | The full text was not available. |
| 78 | Karimi, A [78] | The full text was not available. |
| 79 | Davarpanah, MA [79] | No serology test was used. |
| 80 | Ramezani, A [80] | No serology test was used. |
| 81 | Mansoori, SD [81] | Low quality due to incorrect reporting of prevalence and/or an unclear number of cases with a positive test. |
| 82 | Habibzadeh, S [82] | Low quality due to incorrect reporting of prevalence and/or an unclear number of cases with a positive test. |
| 83 | Khodabakhshi, B [83] | Low quality due to incorrect reporting of prevalence and/or an unclear number of cases with a positive test. |
| 84 | Talebi-Taher, M [84] | The results were pooled across different populations so we could not find population specific data. |
| 85 | Hosseini Asl, SK [85] | The results were pooled across different populations so we could not find population specific data. |
| 86 | Hassan pour, E [86] | The results were pooled across different populations so we could not find population specific data. |

** The article reported co-infections for some subpopulations that we were interested (we included them in analysis) and also other populations.

References

1. Ramezani A, Mohraz M, Gachkar L (2006) Epidemiologic Situation of Human Immunodeficiency Virus (HIV/AIDS Patients) in a Private Clinic in Tehran, Iran. Archives of Iranian Medicine 9: 315-318.

2. Davarpanah M, Darvishi M, Mehrabani D (2007) The Prevalence of HBS antigenemia in patients with HIV infection in Shiraz, southern Iran. Iranian Red Crescent Medical Journal 9: 224-225.

3. Alavi SM, Etemadi A (2007) HIV/HBV, HIV/HCV and HIV/HTLV-1 co infection among injecting drug user patients hospitalized at the infectious disease ward of a training hospital in Iran. Pakistan Journal of Medical Sciences 23: 510-513.

4. Ramezani A, Mohraz M, Banifazl M, Jam S, Gachkar L, et al. (2008) Frequency and associated factors of proteinuria in Iranian HIV-positive patients. International Journal of Infectious Diseases 12: 490-494.

5. Babamahmoodi F, Heidari GM, Mahdi NM, Delavarian L (2012) The prevalence rate of hepatitis B and hepatitis C co-infection in HIV positive patients in Mazandaran province, Iran. Medicinski glasnik: official publication of the Medical Association of Zenica-Doboj Canton, Bosnia and Herzegovina 9: 299-303.

6. Taeri K, Kasaeian N, Nobari RF, Ataei B (2008) The prevalence of hepatitis B, hepatitis C and associated risk factors in intravenous drug addicts (IVDA) with HIV in Isfahan. Journal of Isfahan Medical School 26.

7. Mohammadi M, Talei G, Sheikhian A, Ebrahimzade F, Pournia Y, et al. (2009) Survey of both hepatitis B virus (HBsAg) and hepatitis C virus (HCV-Ab) coinfection among HIV positive patients. Virology journal 6: 202.

8. Ataei B, Tayeri K, Kassaeian N, Farajzadegan Z, Babak A (2010) Hepatitis B and C among patients infected with human immunodeficiency virus in Isfahan, Iran: seroprevalence and associated factors. Hepat Mon 10: 188-192.

9. Sharifi-Mood B, Metanat M (2010) Co-infection HIV/AIDs and Hepatitis C. International Journal of Virology 6: 69-72.

10. Badie BM, SeyedAlinaghi SA, Chaman R, Hosseini M, Hasibi M, et al. (2011) Frequency and Correlates of Co-Infection Hepatitis C and Hepatitis B with HIV. Knowledge & Health 6: 40-43.

11. SeyedAlinaghi SA, Jam S, Mehrkhani F, Fattahi F, Sabzvari D, et al. (2011) Hepatitis-C and hepatitis-B co-infections in patients with human immunodeficiency virus in Tehran, Iran. Acta Medica Iranica 49: 252-257.

12. Rezaianzadeh A, Hasanzadeh J, Alipour A, Davarpanah MA, Rajaeifard A, et al. (2012) Impact of Hepatitis C on Survival of HIV-Infected Individuals in Shiraz; South of Iran. Hepat Mon 12: 106-111.

13. Rahimi MK, Hajighanbari M, Almasi F, Jafari S, Mosavi L, et al. (2007) Epidemiologic survey of Risk factos of 377 HIV+ patients infected Journal of medical Sciences of Azad university 17: 103-106.

14. Bagheri P, Faramarzi H, Sabet M (2011) The Survey of Risk Factors in HIV Positive Patients Covered by Shiraz University of Medical Sciences. Journal of Isfahan Medical School 29: 1341-1349.

15. Keshvari M, Hajarizade B, SM A, Attarchi Z, Hji beigi B, et al. (2004) Prevalence of HBV an HCV and risk factors HIV positive patients who reffered to counseling center Blood Transfusion Organization, Tehran center (2003). 2nd Iranian Congress on Virology.

16. Moniri R, Mosayebii Z, Mossavi G (2004) Seroprevalence of cytomegalovirus, hepatitis B, hepatitis C and human immunodeficiency virus antibodies among volunteer blood donors. Iranian J Publ Health 33: 38-42.

17. Kasraian L, Jahromi T, Ardeshir S (2007) Prevalence of major transfusion transmitted viral infections (HCV, HBV, HIV) in Shiraz blood donors from 2000 to 2005. Scientific Journal of Iran Blood Transfus Organ 3: 373-378.

18. AghaJaniPoor K, Zandieh T (2006) Seroepidemiological investigation of hepatitis B,C and HIV virus in safe blood donors of Babol Blood Transfusion Center. Scientific Journal of Iran Blood Transfus Organ 2: 339-341.

19. Tahaei SME, Mohebbi SR, Azimzadeh P, Vahedi M, Almasi S, et al. (2011) Frequency of HIV and HCV Co-Infections in Chronic HBV Patients Referred to Taleghani Hospital, Tehran, Iran from 2006 to 2010 Hepat Mon 11: 993.

20. Alaoddolehei H, Nourkojori S, Kalantari N, Sadighian F (2010) Effectiveness of confidential self-exclusion (CSE) and failed options on blood donation safety in Sari organization of blood transfusion, 2005. Casp J Intern Med 1: 20-22.

21. Mohammadalizadeh A, Alavian S, Jafari K, Yazdi N (2003) Prevalence of HBS AG, HC AB & HIV AB in the addict prisoners of Hammadan prison (Iran, 1998). Journal of Research in Medical Sciences 7: 311-313.

22. Imani R, Karimi A, Rouzbahani R, Rouzbahani A (2008) Seroprevalence of HBV, HCV and HIV infection among intravenous drug users in Shahr-e-Kord, Islamic Republic of Iran. East Mediterr Health J 14: 1136-1141.

23. Mirahmadizadeh A, Majdzadeh R, Mohammad K, Forouzanfar M (2009) Prevalence of HIV and hepatitis C virus infections and related behavioral determinants among injecting drug users of drop-in centers in Iran. Iranian Red Crescent Medical Journal 11: 325-329.

24. Attarchi Z, Ghafouri M, Hajibaygi B, Assari S, Alavian SM (2006) Donor deferral and blood-borne infections in blood donors of Tehran. Scientific Journal of Iran Blood Transfus Organ 2: 353-364.

25. Mahmoodian-Shooshtari M, Pourfathollah A (2006) An overview analysis of blood donation in the Islamic Republic of Iran. Arch Iranian Med 9: 200-203.

26. Daee Parizi Z, Zahiri N (2006) The prevalence of HBV, HCV and HIV in thalassemia patuents referring to specific diseases center of Kerman during 1999 to 2006 And compare it with the results of 1996. Kerman: Kerman University of Medical Sciences.

27. Emmam J, Assaeh Zadegan M (2004) Seroprevalence of hepatitis B, hepatitis C and HIV in Ahwaz voluntary blood donors. 2nd Iranian Congress on Virology. Tehran.

28. Khedmat H, Alavian SM, Miri SM, Amini M, Abolghasemi H, et al. (2009) Trends in seroprevalence of hepatitis B, hepatitis C, HIV, and syphilis infections in Iranian blood donors from 2003 to 2005. Hepat Mon 9: 24-28.

29. Khodadadizadeh A, Nadimi.A. E, Hossieni S, Shabani Sharbabaki Z (2006) The prevalence of HIV, HBV and HCV in narcotic addicted persons referred to the out patient clinic of rafsanjan university of medical sciences in 2003. Journal of Rafsenjan University of Medical Sciences 5: 23-30.

30. Khumeisipour GR, Tahmasebi R (2001) Infection of HIV, HBV, HCV and syphilis in high risk group , Bushehr, 2000. Iranian South Medical Journal 3: 53-59.

31. Kolivand M, Hashemimehr AS, Safari S (2011) Assessment of HIV prevalence in blood donors in Kermanshah province, 2005. Journal of Kermanshah University of Medical Sciences (J Kermanshah Univ Med Sci) 14.

32. Tofigi H, Ghorbani M, Akhlaghi M, Yaghmaei A, Mostafazadeh B, et al. (2011) Incidence of Hepatitis B and HIV Virus at Cadaver of IV Drug Abusers in Tehran. Acta Medica Iranica 49: 59-63.

33. Torabi S, Abed-Ashtiani K, Dehkhoda R, Moghadam A, Bahram M, et al. (2004) Evaluation frequency of hepatitis B and C and HIV in patients with haemophilia in eastern Azarbaijan province at 2003. Scientific Journal of Iran Blood Transfus Organ 6: 73-82.

34. Rezazadeh M, MANI KKH, Mohammadi A, ZANDVAKILI H, LOTFI A, et al. (2006) Prevalence of human immunodeficiency, hepatitis B and hepatitis C viruses in the first time, repeat and regular donors in blood transfusion center, Hamadan, 2004-2005. Iranian Journal of Infectious Diseases And Tropical Medicine.

35. Rowhani-Rahbar A, Tabatabaee-Yazdi A, Panahi M (2004) Prevalence of common blood-borne infections among imprisoned injection drug users in Mashhad, North-East of Iran. Archives of Iranian Medicine 7: 190-194.

36. S. N-K, H. A, F. S (2007) Efficacy of confidential self-exclusion and failed systems on blood donation safety in Sari and Behshahr blood donors. Scientific Journal of Iran Blood Transfus Organ 4: 153-158.

37. Alavi SM, Behdad F (2010) Seroprevalence study of hepatitis C and hepatitis B virus among hospitalized intravenous drug users in Ahvaz, Iran (2002-2006). Hepatitis Monthly 10: 101-104.

38. Taheri Azbarmi Z, Nouri S, Joukar F, Haji KKH, N.S. A, et al. (2008) Transfusion transmitted diseases in Rasht blood donors. Scientific Journal of Iran Blood Transfus Organ 4: 337-343.

39. Samar G, Amini S, Ebrahimi N, Sadeghi M (1996) survey on HIV, HBV and HCV prevalence in hemophilic and hemodialysis patients and health care workers of hemodialysis centers. 2nd congress of blood and related diseases.

40. A.R. S, M.A. N, M. H, B. K (2008) Transfusion Transmitted Virus prevalence rate in Injection Drug Users (IDUs): a cross sectional study. Tehran University Medical Journal (TUMJ) 66: 282-287.

41. Ebrahimian Z, Fazilati M, Akbari N, Hariri MM, Fatehifar MR (2011) Correlation of deferral rate with the frequency rate of viral markers of HBV, HCV and HIV in blood supplies during 2004 to 2009. Scientific Journal of Iran Blood Transfus Organ 8: 130-136.

42. Shariatzadeh SMA, Naderi GHA (2000) Evaluation infection to HBV, HCV and HIV in patients with thalassaemia major in Markazi province. Oroomieh J Med Sci 11: 20-28.

43. Bani Aghil SS, Abbasi S, Arab M, Seyedein MS (2009) The Prevalence of HCV, HBV, HIV in Blood Donors of Golestan Province, (2006-2008). Medical Laboratory Journal 3: 0-0.

44. Zamani S, Radfar R, Nematollahi P, Fadaie R, Meshkati M, et al. (2010) Prevalence of HIV/HCV/HBV infections and drug-related risk behaviours amongst IDUs recruited through peer-driven sampling in Iran. International Journal of Drug Policy 21: 493-500.

45. Mirahmadizadeh AR, Kadivar MR, Hemmati AR, A. J (2004) Infection with HIV and hepatitis C, and B viruses among injecting drug users in Shiraz, Southern Iran. 15th International Conference on AIDS. Bangkok,Thailand.

46. Abdollahi A, Shahsiah R, Nassiri Toosi M, Lak M, Karimi K, et al. (2008) Seroprevalence of Human Immunodificiency Virus (HIV) and Hepatitis C Infection in Hemophilic Patients in Iran. Iranian Journal of Pathology 3: 119-124.

47. Jahani M, Alavian S, Shirzad H, Kabir A, Hajarizadeh B (2005) Distribution and risk factors of hepatitis B, hepatitis C, and HIV infection in a female population with “illegal social behaviour”. Sexually Transmitted Infections 81: 185-185.

48. Pourazar A., Akbari N., Hariri M., Yavari F., Akbari Sh. (2006) Evaluation of demographic profiles and prevalence of major viral markers in first time vs repeat blood donors in Esfahan. Scientific Journal of Iran Blood Transfus Organ 2: 323-329.

49. Karimi M, Ghavanini AA (2001) Seroprevalence of HBsAg, anti-HCV, and anti-HIV among haemophiliac patients in Shiraz, Iran. Haematologia 31: 251-255.

50. Aghakhani A, Banifazl M, Kalantar E, Eslamifar A, Ahmadi F, et al. (2010) Occult hepatitis B virus infection in hemodialysis patients with isolated hepatitis B core antibody: a multicenter study. Therapeutic Apheresis and Dialysis 14: 349-353.

51. Mahmoudian Shoushtari M, Pourfateh A (2006) An overview analysis of blood donation in the Islamic Republic of Iran. ARCHIVES OF IRANIAN MEDICINE 9: 200-203.

52. Amini Kafi‐abad S, Rezvan H, Abolghasemi H, Talebian A (2009) Prevalence and trends of human immunodeficiency virus, hepatitis B virus, and hepatitis C virus among blood donors in Iran, 2004 through 2007. Transfusion 49: 2214-2220.

53. Esmaeili H, Mankhian AR, Hajiani GR (2013) Efficacy of confidential self-exclusion systems on blood donation safety in Bushehr blood donors. Razi Journal of Medical Sciences 20: 58-63.

54. Khoshnood mansoorkhani MJ, Zand V, Tavakkoli M, Hajarizadeh B, SM A (2003) Survey on Hepatitis C and B in thalassemia patients of Kerman and the role of blood transfution at infection Govaresh 8: 72-78.

55. Hajinasrollah A, Yeganeh R, Salehi N, Saheh M, Khoshkar A, et al. (2006) Prevalence of HIV, Hepatitis B, and Hepatitis C in Drug Abuser in Loghman Medical Center. IJS 13: 89-94.

56. Tabatabaee S, Anbiaei R (1998) Prevalence of HBsAg, HCVAB and HIVAB and their risk factors in hemodialysis patients, Yazd. Yazd: Yazd university of Medical Sciences.

57. Azarkeivan A, Hajibeigy B, Afradi H, Eslami M, Sh. G, et al. (2011) Evaluation of clinical conditions of thalassemic patients having referred to Adult Thalassemia Center, Tehran. Scientific Journal of Iran Blood Transfus Organ 1: 32-41.

58. Moradi A, Khodabakhshi B, Sadeghipour M, Besharat S, Tabarraei A (2011) Concurrent infections of hepatitis C and HIV in hepatitis B patients in the north-east of Iran. Tropical doctor 41: 129-131.

59. Emamghorashi F, Fathi G.H, Mohtashami A (2006) Evaluation of demographic characteristics and Hepatitis B, C and HIV prevalence among blood donors in Jahrom. Scientific Journal of Iran Blood Transfus Organ 2: 373-378.

60. Sayahjoo S, Yaghmaei F (2006) Prevalence of HIV, hepatitis B, C in thalassemia patients, Amir hospital, Semnan. 01st National Air Pollution and its Effects on Health's Seminar. Tehran.

61. Kazeroni P, Allahyari S, Davarpanah M, Ansari G (2006) Seroepidemiological survey of co-inections in HIV+ persons and AIDS patients who were coverd by counseling of behavioral diseases, Shiraz at 1383-1394. Journal of Kermanshah University of Medical Sciences 13: 30.

62. Ghanbarzadeh N, Nadjafi-Semnani M (2006) A study of HIV and other sexually transmitted infections among female prisoners in Birjand. Journal of Birjand University of Medical Sciences 13: 9-15.

63. Kasraian L, Jahromi T (2003) The frequency of HIV infection in blood donors in Shiraz blood transfusion organization from 1998 to 2002. journal of zanjan university of medical sciences and health Services 11: 49-54.

64. Kazerani H (2007) Epidemiologic survey of positive HIV, HCV, HBV tests among patients admitted for cardiac surgery and invasive procedures, In Emam Ali Hospital, in Kermanshah. Scientific Journal of Kurdistan University of Medical Sciences 11: 42-47.

65. Sarveghad MR, Naderi HR, Farokhnia M, Bejdi A (2005) Epidemiological study of injecting drug users admitted in infectious ward of Imam Reza Hospital, Mashhad. Medical Journal of Mashhad University of Medical sciences 48: 79-84.

66. Shariat SV (2004) Prevalence of HBV, HCV and HIV in hemophilic patients of Imam Khomeini Hospital. Tehran: Tehran University of Medical Sciences. 45 p.

67. Sharifi-Mood B, Metanat M (2006) Infection among hospitalized injection drug users. J Med Sci 6: 686-689.

68. Bakhshi S (2003) Frequency of HIV, HBV and HCV infections in dialysis center of SAhahid Beheshti hospital, December 2001-2002. Zanjan: Zanjan University of Medical Science.

69. Meidani M, Farzaneh S, Ajami Baferani A, Hassan Zade A (2009) Seroprevalence of HTLV1, 2 Virus Among Injection Drug Addicts in Isfahan, 2007-2008. SSU_Journals 17: 286-290.

70. Aminzadeh Z, K. AS (2007) Seroepidemiology of HIV, syphilis, hepatitis B and C in intravenous drug users at Loghman Hakim hospital. Iranian Journal of Medical Microbiology 1: 53-56.

71. Sharif M, Sherif A, Sayyah M (2009) Frequency of HBV, HCV and HIV infections among hospitalized injecting drug users in Kashan. Indian Journal of Sexually Transmitted Diseases 30: 28.

72. Keramat F, Eini P, Majzoobi M (2011) Seroprevalence of HIV, HBV and HCV in Persons Referred to Hamadan Behavioral Counseling Center, West of Iran. Iranian Red Crescent Medical Journal 13: 42-46.

73. Vahdani P, Hosseini-Moghaddam SM, Family A, Moheb-Dezfouli R (2009) Prevalence of HBV, HCV, HIV and syphilis among homeless subjects older than fifteen years in Tehran. Arch Iran Med 12: 483-487.

74. Mir-Nasseri MM, Mohammadkhani A, Tavakkoli H, Ansari E, Poustchi H (2011) Incarceration is a major risk factor for blood-borne infection among intravenous drug users: Incarceration and blood borne infection among intravenous drug users. Hepat Mon 11: 19-22.

75. Bagheri-Mahaki E (2004) The study of HBV, HIV, siphilis and toxoplasmosis in injecting drug users: Tehran University of Medical Sciences.

76. Salari Z (2005) Prevalence and risk factors of HIV, HBV and HCV infection in injecting drug users admitted to Imam Khomeini hospital, 1378 through 1384. Tehran: Tehran Univesity of Medical Science.

77. Beheshti M (1999) study on prevalence of HBV and HCV in hemophilia patients of Isfahan city, 1999. Isfahan University of Medical Science.

78. Karimi A Seroprevalence of HBV, HCV and HIV among intravenous drug users in Iran. Journal of Clinical Virology 36: S210-S211.

79. Davarpanah MA, Saberi-Firouzi M, Bagheri Lankarani K, Mehrabani D, Behzad Behbahani A, et al. (2009) Hepatitis C virus genotype distribution in Shiraz, southern Iran. Hepat Mon 9: 122-127.

80. Ramezani A, Banifazl M, Eslamifar A, Aghakhani A (2010) Serological pattern of anti-HBc alone infers occult hepatitis B virus infection in high-risk individuals in Iran. The Journal of Infection in Developing Countries 4: 658-661.

81. Mansoori SD, Zadsar M, Arami S, Adimi S, Alaei K, et al. (2003) Immunological and clinical features of HIV in a group of hospitalized Iranian patients. Archives of Iranian Medicine 6: 5-8.

82. Habibzadeh S, Davarnia B, Bagherzadeh JS, Kholgh G (2005) Epidemiological evaluation of transfusion transmitted diseases in Ardabil in Tasoua and Ashoura 1381 (2003). Scientific Journal of Iran Blood Transfus Organ 1: 55-60.

83. khodabakhshi B, Abbassi A, Fadaee F, Rabiee MR (2007) Prevalence and risk factors of HIV, hepatitis B virus and hepatitis C virus infections in drug addicts among Gorgan prisoners. Journal of Medical Sciences 7: 252-254.

84. Talebi-Taher M, Mohit M, Avanessians TB (2010) Viral hepatitis in patients hospitalized in two teaching hospitals, Tehran, Iran. Iranian Journal of Clinical Infectious Diseases 5: 25-29.

85. Hosseini Asl S, Avijgan M, Mohamadnejad M (2004) High prevalence of HBV, HCV, and HIV infections in Gypsy population residing in Shahr-E-Kord. Arch Iranian Med 7: 20-22.

86. Hasanpour E, Arasth E, Ghorbani S, Mahdavi S (2003) prevalence of hepatits B, hepatitis C and HIV infection in 15 year and older patiants admitted in hand surgery emergency. Scientific Journal of Kurdistan University of Medical Sciences 8: 25-32.
