# Supplementary material for: HIV, HBV and HCV Coinfection Prevalence in Iran - A Systematic Review and Meta-Analysis
Source: PLoS One. 2016 Mar 31;11(3):e0151946. doi: 10.1371/journal.pone.0151946 (PMC4816272; doi:10.1371/journal.pone.0151946)
Supplement: S2 Table — (DOC) [file pone.0151946.s002.doc]

| **Section/topic** | **#** | **Checklist item** | **Reported on page #** |
| --- | --- | --- | --- |
| TITLE | | |  |
| Title | 1 | HIV, HBV and HCV coinfection prevalence in Iran; a systematic review and meta-analysis | Page 1 |
| ABSTRACT | | |  |
| Structured summary | 2 | Background: worldwide, hepatitis C and B virus infections (HCV and HCV), are the two most common coinfections with human immunodeficiency virus (HIV) and has become a major threat to the survival of HIV-infected persons. The review aimed to estimate the prevalence of HIV, HBV, HCV, HIV/HCV and HIV/HBV and triple coinfections in different subpopulations in Iran. **Method:** Following PRISMA guidelines, we conducted a systematic review and meta-analysis of reports on prevalence of HIV, HBV, HCV and HIV coinfections in different subpopulations in Iran. We systematically reviewed the literature to identify eligible studies from January 1996 to March 2012 in English or Persian/Farsi databases. We extracted the prevalence of HIV antibodies (diagnosed by Elisa confirmed with Western Blot test), HCV antibodies and HBsAg (with confirmatory laboratory test) as the main primary outcome. We reported the prevalence of the three infections and coinfections as point and 95% confidence intervals.  **Findings:** HIV prevalence varied from %0.00 (95% CI: 0.00 – 0.003) in the general population to %17.25 (95% CI: 2.94-31.57) in people who inject drugs (PWID). HBV prevalence ranged from % 0.00 (95% CI: 0.00 – 7.87) in health care workers to % 30.9 (95% CI: 27.88 – 33.92) in PWID. HCV prevalence ranged from %0.19 (95% CI: 0.00 – 0.66) in health care workers to %51.46 (95% CI: 34.30 – 68.62) in PWID. The coinfection of HIV/HBV and also HIV/HCV in the general population and in health care workers was zero, while the most common coinfections were HIV/HCV (10.95%), HIV/HBV (1.88%) and triple infections (1.25%) in PWID.  **Conclusions:** We found that PWID are severely and disproportionately affected by HIV and the other two infections, HCV and HBV. Screenings of such coinfections need to be reinforced to prevent new infections and also reduce further transmission in their community and to others. | Page 1-2 |
| INTRODUCTION | | |  |
| Rationale | 3 | HIV and viral hepatitis infections are still the major causes of morbidity and mortality in developing countries, with one billion people directly exposed or at-risk population . Worldwide, 34 million people are infected with HIV, 130 million people are infected with HCV (Hepatitis C Virus), 2 billion people are infected with HBV (Hepatitis B Virus), and 350-400 million people are suffering from viral chronic hepatitis . Annually, approximately two million people die due to AIDS, more than 350 thousands people die from diseases associated with HCV and one million people die as a result of an HBV infection .  In Iran, the prevalence of HIV and other blood-borne viral infections like HCV is relatively low in the general population. Prevention strategies like public awareness on routes of transmission, free HIV testing and counseling services at public health facilities and correctional institutes like prisoners contributed to this low prevalence. Screening for HCV and HIV in all blood donors and all blood products have been in place since 1996 and 1989 respectively. Countrywide harm reduction services including, but not limited to needle exchange programs (delivered by 682 centers ) and drug treatments like methadone maintenance therapy (delivered by 4275 centers) have been implemented by governmental funds . The government of Iran is committed to provide universal access to HIV prevention (free condom, education, HIV testing) and antiviral therapy services for all at-risk or affected populations as outlined in the 4th National AIDS Strategic Plan 2015-19. These heath policies and interventions aim to reduce the burden of main blood-borne infections in Iran.  HCV and HIV share common transmission risk behaviors, either monoinfection or HCV/HIV coinfection have been reported in population of drug injectors worldwide . Despite HIV and HBV, sexually-acquired or vertical transmitted HCV is not common . These coinfections could lead to accelerated chronic hepatitis and liver cancer , which reported as one of the major causes of morbidity and mortality in HIV-infected individuals . The most affected population are PWID .  The HIV epidemic in Iran is concentrated among PWID with the pooled HIV prevalence of 18.4% (95% CI: 16.7, 20.2) after 2005. HIV has been in the radar of national AIDS prevention and treatment programs. The trends of HIV and risk behaviors have been studied in several national bio-behavioral surveys . However, screening for HCV in HIV-infected patients and annual screening in high-risk population like PWID, as recommendations by guidelines , has not been implemented systematically. One reason is that the scope of HCV and HBV coinfections with HIV has not been studied in Iran.  Many subnational studies have assessed HIV and HCV coinfections, mostly among people who inject drugs , and prisoners , however the overall size of such co-epidemics is unknown in Iran. | Pages 2, 3 |
| Objectives | 4 | In this systematic review, we aimed to estimate the prevalence of HIV, HCV and HBV infection and HIV coinfections and identify the most affected subpopulations in Iran. | Page 3 |
| METHODS | | |  |
| Protocol and registration | 5 |  |  |
| Eligibility criteria | 6 | Only studies that recruited participants living in Iran, published in Persian/Farsi or English, measured HIV (HIV Ab) and coinfections such as HBV (HBs Ag) or HCV (HCV Ab) infection with confirmatory lab tests were included. We excluded studies with 1) no accessible full text and no sufficient data in abstract, 2) unclear serological tests to detect the three infections, 3) low quality due to incorrect reporting of prevalence and/or an unclear number of cases with a positive test and 4) reported viral hepatitis prevalence only in HIV-positive individuals (i.e. only HIV positive cases were recruited in the study). | Page 4 |
| Information sources | 7 | Between March and June 2012 we searched multiple English and Persian/Farsi electronic data sources including Pubmed, Iranmedex, Google Scholar, Iranian Data Bank of Hepatitis Research, Iranian Data Bank of HIV Research, Scientific Information Database (SID), Magiran and the Iran Blood Transfusion Journal. We hand-searched the references and selected the relevant articles for inclusion. We also looked at the electronic abstract list of congresses conducted in Iran and also at the electronic database of students’ thesis through universities’’ electronic libraries and websites, when was available. | Page 4 |
| Search | 8 | Keywords that we used for our search were “HIV and HBV", "HIV and HCV", "viral hepatitis and HIV" and "coinfection and HIV". | Page 4 |
| Study selection | 9 | We reviewed the titles and abstracts to select potentially relevant papers. Following this screening process, we reviewed the abstract of the papers. If there was doubt about the suitability of the paper based on the abstract alone, the full text was reviewed and If the full text was not accessible, it was grouped as excluded study. | Page 4, S1 table |
| Data collection process | 10 | Data was extracted by one reviewer and double checking of studies. | Page 4-5 |
| Data items | 11 | Information was extracted as following: type of study, sample size, location and time of the study, type of participants and prevalence of HIV, HBV and HCV and the coinfections | Page 4-5 |
| Risk of bias in individual studies | 12 |  |  |
| Summary measures | 13 | We conducted meta-analyses in STATA version 11. We did meta-analysis for each HIV, HCV, HBV, HIV/HBV, HIV/HCV and HIV/HBV/HCV prevalence in every subpopulation, pending on the data availability. The outcome was reported as prevalence, with point and 95% confidence intervals. A Q-test was used to assess heterogeneity. When the heterogeneity test was statistically significant (p-value < 0.1), a random-effects model was used; otherwise the fixed-effects model was applied to calculate the pooled prevalence. | Page 5 |
| Synthesis of results | 14 | Pooled estimate were calculated for HIV, HBV, HCV, HIV/HBV, HIV/HCV and HIV/HBV/HCV in each subpopulations | Page 5 |

Page 1 of 2

| Section/topic | # | Checklist item | Reported on page # |
| --- | --- | --- | --- |
| Risk of bias across studies | 15 |  |  |
| Additional analyses | 16 |  |  |
| RESULTS | | |  |
| Study selection | 17 | As presented in Figure 1, we found 302 abstracts in our literature review. After removing duplications (167) based on title and abstract, 135 remained for fulltext review. Of those, 83 articles were excluded (S1 table) for various reasons and 49 study were remained. | Page 6 |
| Study characteristics | 18 | See table 1 | Pages 11-12 |
| Risk of bias within studies | 19 |  |  |
| Results of individual studies | 20 |  |  |
| Synthesis of results | 21 | HIV infection: The HIV prevalence in different subgroups varied from %0.00 (95% CI: 0.00-0.003) in the general population to %17.25 (95% CI: 2.94-31.57) in PWID. Prisoners were the second affected group, following PWID, with HIV prevalence as high as 3.42% (95% CI: 1.22-5.63). HIV prevalence in other groups was very low and close to zero.  HBV infection: The prevalence of HBV varied from %0.00 (95% CI: 0.00-7.87) in health care workers to the highest level of %30.9 (95% CI: 27.88-33.92) in PWID. HBV prevalence in the general population was close to 1%, and in PWRMT near to 0.7%.  HCV infection: The prevalence of HCV was surprisingly high among PWID as 51.46% (95% CI: 34.30-68.62). In addition, 34.73% of prisoners diagnosed with HCV, followed by 19.28% in PWRMT. HCV was assessed among street children in two studies with an estimated prevalence of 0.76%, considerably higher than the prevalence in the general population (0.31%).  HIV and HBV coinfection: Prevalence of both HBV and HIV in the general population, health care workers and PWRMT was very low and close to 0%. The highest prevalence of HIV/HBV coinfection was observed among injecting drug users as 1.88% (95% CI: 0.00-4.03). Among the prisoners, the HIV/HBV prevalence (0.13%) was lower than the PWID group but more than the other three subpopulations.  HIV and HCV coinfection: The HIV/HCV coinfection prevalence was very low and close to zero among the general population, health care workers and street children. However, 10.95% (95%CI: 2.82-19.08%) of PWID were positive for both HIV and HCV. In compare to general population, prisoners had also a higher prevalence of HIV/HCV (1.71%, 95%CI 0.11-3.30).  HCV/HBV/HIV coinfection: The prevalence of such triple coinfections was very low and close to zero in the general population, health care workers and street children; while it peaked to %1.25 (95% CI: 0.00-3.01) in PWID. PWRMT and prisoners had a low prevalence as 0.01% and 0.28%, not statistically different from the prevalence in the general population. | Pages 6,7 |
| Risk of bias across studies | 22 |  |  |
| Additional analysis | 23 |  |  |
| DISCUSSION | | |  |
| Summary of evidence | 24 | We found that PWID are severely and disproportionately affected by HIV and the other two infections, HCV and HBV. Prisoners are the second population who have high prevalence of hiv- co infections Screenings of such coinfections need to be reinforced to prevent new infections and also reduce further transmission in their community and to others. | Pages 8-10 |
| Limitations | 25 | We only include studies that have reported joint probabilities of coinfections. Age was reported very differently by the original studies, so we were not able to report the findings by age groups. | Page 10 |
| Conclusions | 26 | Our results highlighted the seriousness of viral hepatitis, particularly HCV, as a coinfection with HIV. Half of people who inject drug affected by HCV while one in four affected by HIV. Given the current HIV/HCV syndemicity in Iran, joint planning, surveillance, healthcare delivery, disease prevention, and clinical care delivery can help to reduce the burden of these infection in Iran more effectively. | Page 10 |
| FUNDING | | |  |
| Funding | 27 | The authors have no support or funding to report. | Page 10 |

*From:*  Moher D, Liberati A, Tetzlaff J, Altman DG, The PRISMA Group (2009). Preferred Reporting Items for Systematic Reviews and Meta-Analyses: The PRISMA Statement. PLoS Med 6(6): e1000097. doi:10.1371/journal.pmed1000097

For more information, visit: **www.prisma-statement.org**.

Page 2 of 2
